# The limits of stress-tolerance for zooplankton resting stages in freshwater ponds

**DOI:** 10.1007/s00442-023-05478-8

**Published:** 2023-11-16

**Authors:** Joana L. Santos, Dieter Ebert

**Affiliations:** Department of Environmental Sciences, Zoology, University of Basel, Vesalgasse 1, 4051 Basel, Switzerland

**Keywords:** Diapause, Temperature, Drought, Hatching success, Local adaptation

## Abstract

In seasonal environments, many organisms evolve strategies such as diapause to survive stressful periods. Understanding the link between habitat stability and diapause strategy can help predict a population’s survival in a changing world. Indeed, resting stages may be an important way freshwater organisms can survive periods of drought or freezing, and as the frequency and extent of drought or freezing vary strongly among habitats and are predicted to change with climate change, it raises questions about how organisms cope with, and survive, environmental stress. Using *Daphnia magna* as a model system, we tested the ability of resting stages from different populations to cope with stress during diapause. The combination of elevated temperatures and wet conditions during diapause shows to prevent hatching altogether. In contrast, hatching is relatively higher after a dry and warm diapause, but declines with rising temperatures, while time to hatch increases. Resting stages produced by populations from summer-dry habitats perform slightly, but consistently, better at higher temperatures and dryness, supporting the local adaptation hypothesis. A higher trehalose content in resting eggs from summer-dry habitat might explain such pattern. Considering that temperatures and summer droughts are projected to increase in upcoming years, it is fundamental to know how resting stages resist stressful conditions so as to predict and protect the ecological functioning of freshwater ecosystems.

**Supplementary Information:**

The online version contains supplementary material available at 10.1007/s00442-023-05478-8.

## Introduction

To overcome environmental fluctuations and seasonal conditions that generate temporarily adverse environments, many organisms have evolved strategies to escape in space (e.g., migration) or time (e.g., dormancy) (Bauer and Hoye 2014; Iwaya-Inoue et al. 2018; Shatilovich et al. 2023). Diapause, a form of dormancy that is characterized by the slow-down or even arrest of biological activities such as development and growth, is expressed in anticipation of severe environmental conditions (Renfree and Shaw 2020; Hand et al. 2016). During diapause, organisms rely on dormant cysts, gemmules, eggs or larvae capable of surviving extreme conditions such as cold, heat and desiccation (Renfree and Shaw 2020). Many prokaryotes (Potts 1994) and eukaryotes, including plants (Hoekstra et al. 2001; Ambastha and Tiwari 2015), fungi (Gadd et al. 1987), and animals (Wright 2001; Kikawada et al. 2005), survive extreme temperatures and desiccation (even when 99% of the water is removed from their cells) (Crowe et al. 1992; Alpert 2005) by producing specific molecules such as sugars (e.g., sucrose and trehalose) (Koster and Leopold 1988; Hoekstra et al. 2001; Tapia and Koshland 2014) and small stress proteins (e.g., heat shock and late embryogenesis abundant proteins) (Somero et al. 2017; Janis et al. 2018). However, the production of these molecules, and dormancy itself, seems to be energetically costly (Wiemken 1990; Ellers and Van Alphen 2002; Moraiti et al. 2023), suggesting that organisms face a trade-off between investment in diapause and other life-history traits.

Across a species’ geographic range, the likelihood of extreme environmental conditions may vary strongly, often correlated with the local climate. Because habitat variation can result in local adaptation and genetic differentiation among populations (Kawecki and Ebert 2004; Wang et al. 2022), a species’ investment in resting stages will thus differ across populations. Indeed, dormancy can be shaped by local adaptation in plants (Kronholm et al. 2012; Price et al. 2018; Wyse and Dickie 2018), insects (Moraiti et al. 2014; Pruisscher et al. 2018; Erickson et al. 2020) and crustaceans (Barata et al. 1995; Roulin et al. 2013, 2015; Radzikowski et al. 2018). In freshwater ecosystems, summer in some populations may coincide with extreme temperatures and desiccation (Seefeldt and Ebert 2019), whereas winter freezing may cause stress in other, geographically distinct populations. Resting stages of freshwater organisms may evolve specific adaptations and strategies to deal with the local conditions and increase survival. For example, a planktonic crustacean from habitats with a high propensity to dry-up in summer has been shown to have elevated levels of trehalose (Santos and Ebert 2022), a sugar known to protect cells from rupture during desiccation (Crowe et al. 1998; Tapia and Koshland 2014). This study by Santos and Ebert (2022) suggests that investment in stress-resistance may entail costs, thus only evolving in populations and habitats where the benefits outweigh those costs.

The planktonic crustacean *Daphnia magna* is an ideal organism to study local adaptation in diapause. It inhabits a wide variety of brackish and freshwater habitats ranging from permanent ponds and lakes to more temporary habitats that dry in summer and/or freeze in winter (Roulin et al. 2013; Seefeldt and Ebert 2019; Ebert 2022), and it produces diapausing resting stages to ensure its survival during conditions unsuitable for its planktonic phase (Kleiven et al. 1992; Brendonck and De Meester 2003; Roulin et al. 2013). Using *D. magna* as a model, we explore how resting stages cope with extreme conditions during diapause by exposing field-collected and laboratory-produced resting stages to different elevated temperatures and wet or dry conditions, and then estimating hatching success and time to hatch after induction. Resting stages undergoing a harsh diapause (i.e., drought, heat) may require specific energetic resources and more time to re-start their development, resulting in decreased hatching success and increased time to hatch. Furthermore, we look at how the combined effects of elevated temperature and desiccation influence the hatching success of resting stages from locations with distinct habitat conditions: summer-dry versus summer-wet waterbodies. We hypothesize that populations adapt locally, such that hatching success after a hot, dry treatment is higher for resting stages from summer-dry habitats than from summer-wet habitats. Lastly, as trehalose content in resting eggs is variable among habitats (Santos and Ebert 2022), we aim to understand whether higher hatching rates are linked to higher trehalose content.

## Methods

### Study system

*Daphnia magna* is a freshwater planktonic crustacean widely distributed across the Holarctic (e.g., Ebert 2022). It reproduces mainly through cyclical parthenogenesis, switching to sexual reproduction when triggered by the environment (e.g., photoperiod, temperature, food) (Kleiven et al. 1992; Roulin et al. 2013). Sexual reproduction requires a synchrony between sexual ratio adjustment, with male offspring production, and the subsequent formation, fertilization and maturation of sexual eggs (Kleiven et al. 1992). The ephippium encapsulates up to two resting eggs (i.e., sexual eggs, specifically embryos with an arrested development), which are the ones that undergo diapause. The duration of diapause is variable among genotypes, but seasonal fluctuations in environmental conditions, such as photoperiod and temperature, are a trigger for its induction, as well as its termination and the restart of development (Stross 1966; Brendonck and De Meester 2003). In nature, these resting stages (i.e., ephippia with resting sexual eggs) are essential for survival, mainly when conditions are unsuitable for the planktonic phase, such as when habitats tend to dry or freeze, so populations are induced to produce sexual resting stages prior to the dry or freezing season, and these stages only hatch when environmental conditions are again adequate (Brendonck and De Meester 2003). Resting stages also migrate passively, transported by wind or animals (e.g., birds) (Alfsnes et al. 2016), thus contributing to a population’s genetic diversity when older resting stages from deeper sediments hatch (Brendonck and De Meester 2003).

### Description of experiments

We conducted four experiments to study how elevated temperatures combined with wet or dry diapause conditions influence hatching success (i.e., “success” or “failure” of hatching per sexual resting egg) and time to hatch (i.e., number of days a resting egg requires to hatch after induction) in *D. magna* resting stages from distinct populations. For the first three experiments, we used resting stages collected from natural pond sediments of different populations. They were thus produced in the field population following natural environmental cues and are likely a result of outbreeding crosses reflecting population dynamics (later addressed in the discussion). These experiments exposed the resting stages to different diapause treatments (wet versus dry) and/or lengths of the diapause treatments, allowing us to determine the consistency of the tested effects and any variation between habitat types. Since field-collected resting stages do not allow us to control for maternal and environmental effects, and are of unknown age duration, we conducted a fourth experiment, subjecting laboratory-produced resting stages subjected to similar diapause conditions. This experiment also used a larger number of genotypes from distinct habitat types to explore the difference between summer-dry and summer-wet habitat types.

#### Resting stages origin

For the three first experiments, sediment samples were collected from natural ponds in Finland (FI-HA1-1, GPS coordinates: N59.842, E23.258), Germany (DE-K2-2; N48.206, E11.706) and Switzerland (CH-H; N47.558, E8.862) or Iran (IR-GG; N37.919, E46.707) (*see* below detailed description for each experiment). The Finnish and Iranian locations represent a summer-dry habitat, while the German and Swiss sites are summer-wet habitats (Roulin et al. 2013; Seefeldt and Ebert 2019). In *Daphnia,* resting stages can survive up to several years in pond sediments (*see* Brendonck and De Meester 2003). Storage in dark, cold conditions can prolong diapause and seems to be fundamental for higher hatching rates in certain populations (De Meester 1993; Stross 1966). The wet sediment samples used in this study were collected at variable times, and stored in the dark at 4 °C for a period between six months (Swiss population) and 20 years (German population). Resting stages were collected by filtering the mud using a 250 µm net with deionized water, placing it in a petri-dish, and carefully collecting *D. magna* resting stages with a stereomicroscope and forceps.

To control for maternal, environmental and age effects, we produced resting stages under controlled laboratory conditions for a fourth experiment. Forty-four genotypes (genetically identical lines) were selected from the *D. magna* Diversity Panel (Yampolsky et al. 2014; Fields et al. 2015; Seefeldt and Ebert 2019)—a collection of *D. magna* genotypes maintained in the laboratory by asexual reproduction. Each chosen genotype originated from a different population across summer-dry and summer-wet habitats in the western Palearctic region (Supplementary Table S1). For each genotype, we reared and clonally reproduced replicate populations in 360-mL jars in ADaM, feeding each clonal population three times a week with 50 Mio cells of *Tetradesmus obliquus* (Wynne and Hallan 2015) algae suspension. Because male and resting stage production is stimulated by short day lengths and high density in certain genotypes (Kleiven et al. 1992; Roulin et al. 2013), we incubated all genotypes in a climate chamber with two different conditions: 20 °C with a 16 h/8 h day/night cycle, and 16 °C with a 12 h/12 h day/night cycle. Replicate populations were allowed to produce fertilized resting stages over 12 months under these conditions. Every four weeks, the populations were transferred to a new jar with fresh ADaM, and resting stages were collected in a closed 100-mL jar with ADaM. As mentioned above, diapause termination is brought about by shifts in environmental conditions. Therefore, to avoid early hatching, we kept the resting stages in the same conditions as the populations in which they were produced over the 12-month period (no hatchlings were recorded during this time). After that time period, all resting stages were placed in 1.5-mL Eppendorf tubes filled with *Daphnia* medium (ADaM) (Klüttgen et al. 1994), and all tubes were together stored in the dark at 4 °C for three months.

The viability of the sexual resting eggs is difficult to determine without risking damage to the resting stages. Indeed, the ability to observe and characterize the eggs depends on the characteristics of the ephippia, such as colour, transparency and rigidity, which vary among populations and genotypes (e.g., Gerrish and Cáceres 2003). Therefore, about 20 resting stages per population/genotype were opened to evaluate the general pattern of the eggs, before the experiments. In general, viable eggs are shiny and greenish-yellow, with a full oval shape whereas old eggs may look more brownish (although they can still be viable). Dead resting eggs have often lost their oval shape and turned pale-yellow in colour. However, no clear definition determines the potential viability of the eggs. Generally, all eggs looked viable, with only few exceptions.

#### Standard protocol for diapause treatments and resting stages hatching

After the initial diapause under dark, cold (4 °C) conditions, the resting stages for all experiments were exposed to different diapause treatments. For wet diapause treatments, resting stages were kept in a closed 1.5-mL Eppendorf tube with ADaM and placed in an incubator or thermomixer for the designated time at the desired temperature (*see* below for each experiment’s details). For dry treatments, the medium from the Eppendorf tubes was removed using a pipette, and the tubes remained open during the diapause treatments. Because bleach solution increases the response of resting stages to light stimulus, and hatching is higher at 20–25 °C (Stross 1966; Haghparast et al. 2012), all resting stages were cooled to room temperature after the diapause treatment period and treated with a 2.5% bleach solution for 5 min to stimulate hatching, followed by a rinse with deionized water for one minute (Retnaningdyah and Ebert 2012). Then, resting stages were placed in a ball-shaped metal mesh container (a tea infuser) in a jar with ADaM at 20 °C and a 16 h/8 h day/night cycle. The tea infuser material has no impact on the resting stages’ hatching or survival; its purpose is to prevent the previously dried resting stages from floating to the water’s surface. The first *D. magna* hatchlings were usually observed three to four days after induction, with hatching peaking over the next two to four days and then continuing in low numbers for up to 20 days (De Meester 1993; Retnaningdyah and Ebert 2012; Radzikowski et al. 2018). Since the number of resting eggs within each ephippia is variable (from 0 to 2), we quantified hatching success by opening all resting stages at the end of the experiment to count the number of non-hatched eggs. The vast majority of the resting eggs appeared viable.

#### Experiment 1

**Fig. 1:**
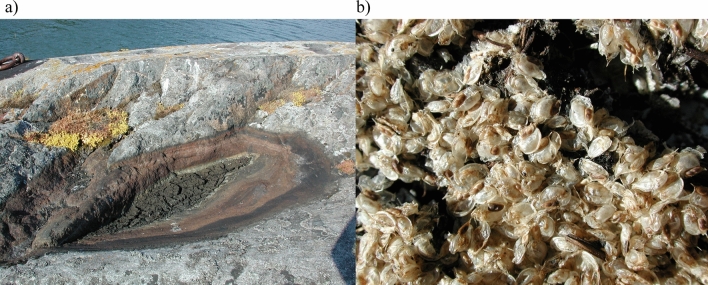
Characteristic summer-dry habitat of *Daphnia magna* populations. Rock pool in Finland (FI-HA1-1, GPS coordinates: N59.842, E23.258) in July (**a**) with *Daphnia magna* resting stages dry and exposed to the sun (**b**)

Using *D. magna* resting stages collected from the summer-dry Finnish population (FI) (N = 646), and the summer-wet German (DE) (N = 630) and Swiss (CH) (N = 704) populations, we designed diapause treatments using a full factorial design of wet and dry conditions combined with elevated temperatures (40, 50, 60, 70 and 80 °C) (Table 1). We also did a treatment at 20 °C, which functions as a control in laboratory experiments (Table 1). Natural populations are likely to experience elevated temperatures (sediment surfaces in summer-dry ponds have been reported to reach almost 50 °C (Seefeldt and Ebert 2019)—but may even go higher in sun exposed and dark places (Zhao et al. 2021) (see Fig. 1)). Note that the study by Seefeldt and Ebert (2019), however aimed to measure water temperature, not sediment temperature). The diapause treatments lasted for 20 days with the first and the last day at 20 °C. Per treatment and population, we used three technical replicates of 30 resting stages each (a total of 90 resting stages with two potential sexual resting eggs each). After hatching induction (*see* section above), hatchlings were recorded and removed from replicate jars daily for 20 days.

**Table 1:**
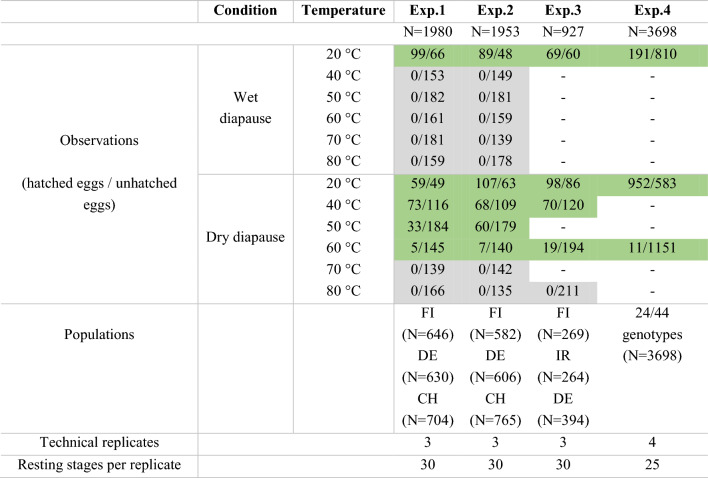
Schematic representation of experiments 1 to 4

Description of the experiments used in this study: overall sample size (N), diapause treatments (Condition & Temperature) with the total observations (i.e., the sum of hatched and unhatched resting eggs), populations used, and of the number of resting stages per technical replicate. Note that each resting stage can have up to two resting eggs. Green-shading represents treatments where at least one hatchling was observed; grey-shading represents treatments without any hatchlings; “-” indicates diapause treatments not staged in that experiment. Populations codes stand for summer-dry populations of FI – Finland (FI-HA1-1, GPS coordinates: N59.842, E23.258) and IR- Iran (IR-GG, N37.919, E46.707), and summer-wet populations of DE – Germany (DE-K2-2, N48.206, E11.706) and CH – Switzerland (CH-H, N47.558, E8.862). In experiment 4, only 24 of 44 genotypes were used for data analysis, due to lack of hatchings at 20 °C, with 13 and 11 genotypes from summer-dry and summer-wet habitats, respectively

#### Experiment 2

Experiment 2 was similar to experiment 1, using naturally produced resting stages from the same three populations (FI (N = 525), DE (N = 606) and CH (N = 765)) as well as the same number of technical replicates and resting stages per replicate and the same diapause treatment combinations (full factorial design: wet/dry conditions crossed with six temperatures) (Table 1). However, to establish the consistency of the results and ensure that they were not the result of a very intense diapause, we shortened the diapause treatments from 20 to 11 days: 2 days at 20 °C, 7 days at the respective temperature treatment, and again 2 days at 20 °C. Hatching trials took place as described for experiment 1.

#### Experiment 3

In experiment 3, we used naturally produced resting stages from three populations, specifically a new summer-dry population from Iran (IR) (N = 264) along with the two previously used populations from Finland (FI, summer-dry) (N = 269) and Germany (DE, summer-wet) (N = 394) (Table 1). The experiment followed the same protocol as above using the same number of technical replicates and resting stages; however, given that no resting stages hatched in the first two experiments from wet, heated diapause treatments, we conducted only one wet diapause treatment at 20 °C and four dry treatments at 20, 40, 60 and 80 °C each (Table 1). Diapause treatments lasted 15 days, with the first and last two days at 20 °C. Hatching trials took place as described for experiment 1.

#### Experiment 4

In experiment 4, we used laboratory-produced resting stages. For each *D. magna* genotype and treatment combination in the experiment, we used four technical replicates with 25 resting stages each (Table 1, Table SS1). Based on the previous experiments’ results and the lack of sufficient resting stages for some *D. magna* genotypes, we set up only three treatments: a wet treatment at 20 °C, and dry treatments at 20 °C and 60 °C, so as to test the hypothesis we observed in previous experiments: that in a dry treatment at 60 °C, only resting stages produced by genotypes from summer-dry habitats would hatch (Table 1). In total, 300 resting stages were used per genotype (25 resting stages × 3 treatments × 4 technical replicates; N = 3698). The diapause treatment lasted 15 days, with the first and last two days at 20 °C, and the subsequent hatching trials occurred as described for experiment 1.

### Data analysis

Data analysis was performed in R studio v1.3.1073 (RStudio Team 2020) with R v4.0.2 (R Core Team 2020) using the packages ggplot2 (Wickham et al. 2020), plyr v1.8.7 (Wickham 2022a), dplyr v1.0.7 (Wickham et al. 2021), tidyr v1.1.4 (Wickham 2021), stringr v1.4.0 (Wickham 2022b) and cowplot v1.1.1 (Wilke 2020) for data handling and presentation. For all four experiments, analysis focused on hatching success (binomial observation—“success” or “failure”—of hatching per sexual resting egg) and time to hatch (days until hatching after induction per hatchling) as dependent variables. Hatching success took into account sexual resting eggs that remained in their ephippia (i.e., that failed to hatch), whereas time to hatch considered only treatments with successfully hatched resting eggs (Table 1). To present the data, we used hatching percentage and days until hatching for each technical replicate. For experiment 4, those averaged values were grouped per habitat type. Each experiment was analysed separately using a Generalized Linear Mixed Model (GLMM) and applying the *glmer* function from the lme4 v1.1–23 package (Bates et al. 2015). For hatching success, a binomial error term distribution was applied due to the discrete distribution of the data based on a success and failure response. Overdispersion was rejected in the model for each experiment (using DHARMa V0.4.6; http://florianhartig.github.io/DHARMa/). For time to hatch, a Poisson error distribution was used. This model was selected based on the evaluation of the data distribution using the *descdist* and *fitdist* functions for discrete data, from the fitdistrplus v1.1–11 package (Delignette-Muller and Dutang 2015). We also compared different fitted models and tested for overdispersion using the additional R packages MASS v5.3–54 (Venables and Ripley 2002) and DHARMa V0.4.6 (http://florianhartig.github.io/DHARMa/) (see Supplementary Fig. SS1 for more details on model selection for time to hatch). The GLMM model included temperature, condition (dry/wet treatment), and habitat type (summer-dry or summer-wet) as explanatory variables, and their interaction as fixed factors. Population/genotype (nested in habitat) and technical replicate (nested in population/genotype) were treated as random factors. Models can be described as “Hatching success (1/0) or Time to hatch (days) ~ Temperature * Condition * Habitat-type + (1| Habitat: Population) + (1| Habitat: Population: Technical replicate)” (see Table 2). In experiment 3 and 4 for hatching success, and in all experiments for time to hatch, the interaction between temperature and condition was not included in the model because it did not fit the experimental design (Table 1). The significance of fixed factors and their interactions were assessed using the *Anova* function of the car package (v3-0–11) (Fox et al. 2021). In experiment 4, we used the *lmer* function from the lme4 v1.1–23 package (Bates et al. 2015) on wet and dry diapause treatments at 20 °C (there were hardly any hatchlings at the higher temperature) to estimate the total variance explained by genotype as random effect. Since only 24 of the 44 *D. magna* genotypes used in experiment 4 hatched successfully in the 20 °C wet/dry diapause treatment, our analysis focused exclusively on those genotypes (13 genotypes from summer-dry habitats and 11 genotypes from summer-wet habitats) (Table SS1).

**Table 2:** Main results from two distinct Generalized Linear Mixed Models (GLMM) assessing the effect of temperature, condition, habitat type and their interactions on hatching success (binomial error distribution) and time to hatch (Poisson error distribution)

Variables	General outcome	Exp.1	Exp.2	Exp.3	Exp.4
Hatching success		(N = 1980)	(N = 1953)	(N = 927)	(N = 3698)
Temperature	Declines with increasing temperature	**Yes**	**Yes**	**Yes**	**Yes**
		χ^2^ = 73.85 *p* < 2.2 × 10^–16^	χ^2^ = 156.85 *p* < 2.2 × 10^–16^	χ^2^ = 80.80 *p* < 2.2 × 10^–16^	χ^2^ = 150.88 *p* < 2.2 × 10^–16^
Condition	Wet versus dry	wet < dry	**wet < dry**	wet > dry	**wet < dry**
		Df = 2 χ^2^ = 5.03 *p* = 0.081	Df = 2 χ^2^ = 11.72 *p* = 0.003	χ^2^ = 0.45 *p* = 0.503	χ^2^ = 95.10 *p* < 2.2 × 10^–16^
Habitat	Habitat type with higher hatching success	**SW**	**SD**	**SD**	SD
		Df = 2 χ^2^ = 9.26 *p* = 0.009	Df = 2 χ^2^ = 21.13 *p* = 2.6 × 10^–5^	χ^2^ = 17.29 *p* = 3.2 × 10^–5^	χ^2^ = 0.07 *p* = 0.798
Temperature * Condition		χ^2^ = 0.0005 *p* = 0.983	χ^2^ = 0.0002 *p* = 0.989	–	–
Temperature * Habitat		χ^2^ = 0.21 *p* = 0.647	χ^2^ = 0.16 *p* = 0.693	χ^2^ = 0.02 *p* = 0.901	χ^2^ = 0.0008 *p* = 0.977
Condition * Habitat	Habitat type with higher hatching success at dry conditions	**SD**	**SD**	SD	**SD**
		χ^2^ = 4.43 *p* = 0.035	χ^2^ = 10.35 *p* = 0.001	χ^2^ = 1.38 *p* = 0.240	χ^2^ = 8.33 *p* = 0.004
Temperature * Condition * Habitat		χ^2^ = 0 *p* = 1.00	χ^2^ = 0 *p* = 1.00	–	–
Population		χ^2^ = 0.028 *p* = 0.868	χ^2^ = 0 *p* = 1	χ^2^ = 0 *p* = 1	χ^2^ = 45.83 *p* = 1.3 × 10^–11^
Technical replicate		χ^2^ = 35.33 *p* = 2.8 × 10^–9^	χ^2^ = 4.76 *p* = 0.029	χ^2^ = 10.12 *p* = 0.001	χ^2^ = 179.23 *p* < 2.2 × 10^–16^

Time to hatch		(N = 269)	(N = 331)	(N = 256)	(N = 1154)
Temperature	Increases with increasing temperature	**Yes**	**Yes**	**Yes**	**Yes**
		Df = 3 χ^2^ = 10.74 *p* = 0.013	Df = 3 χ^2^ = 11.09 *p* = 0.011	Df = 2 χ^2^ = 52.05 *p* = 5.0 × 10^–12^	χ^2^ = 22.92 *p* = 1.7 × 10^–6^
Condition	Wet versus dry	**wet < dry**	**wet < dry**	**wet < dry**	**wet < dry**
		χ^2^ = 15.54 *p* = 8.1 × 10^–5^	χ^2^ = 14.34 *p* = 1.5 × 10^–4^	χ^2^ = 37.21 *p* = 1.1 × 10^–9^	χ^2^ = 3.96 *p* = 0.047
Habitat	Habitat type with higher time to hatch	SD	**SW**	**SW**	SD
		χ^2^ = 1.80 *p* = 0.180	χ^2^ = 4.21 *p* = 0.040	χ^2^ = 48.37 *p* = 3.5 × 10^–12^	χ^2^ = 1.95 *p* = 0.162
Temperature * Habitat		Df = 2 χ^2^ = 0.98 *p* = 0.612	Df = 2 χ^2^ = 0.78 *p* = 0.678	χ^2^ = 0.79 *p* = 0.375	–
Condition * Habitat		χ^2^ = 0.07 *p* = 0.788	χ^2^ = 0.003 *p* = 0.954	χ^2^ = 2.72 *p* = 0.099	χ^2^ = 0.02 *p* = 0.885
Population		χ^2^ = 9.628 *p* = 0.002	χ^2^ = 6.55 *p* = 0.010	χ^2^ = 0 *p* = 1	χ^2^ = 87.18 *p* < 2.2 × 10^–16^
Technical replicate		χ^2^ = 1.51 *p* = 0.220	χ^2^ = 0 *p* = 1	χ^2^ = 0 *p* = 1	χ^2^ = 0 *p* = 0.999

The random effects of population (experiment 1 to 3) or genotype (experiment 4) were nested in habitat, whereas the random effects of technical replicate were nested in population/genotype. If the outcome for a tested variable is highlighted in bold, it indicates that it is significant at a *p* value < 0.05. “N” stands for sample size; “SW” and “SD” correspond to summer-wet and summer-dry habitats, respectively; Df, χ^2^ and *p* stand for model statistics of degrees of freedom (if not shown tests had Df = 1), chi-squared and *p*-value, respectively

### Hatching success and trehalose content

A previous study measured the trehalose content per dry weight of resting eggs from diverse genotypes (Santos and Ebert 2022). For 21 samples from experiment 4 (*see* Table SS1), we used these genotype-specific trehalose estimates to test for a correlation between trehalose content and hatching success, after the 20 °C dry (N = 1305) and wet diapause (N = 807) treatments separately, using the *lm* function. This analysis was not performed for the 60 °C, dry treatment, since most genotypes failed to hatch after that diapause treatment.

## Results

**Fig. 2:**
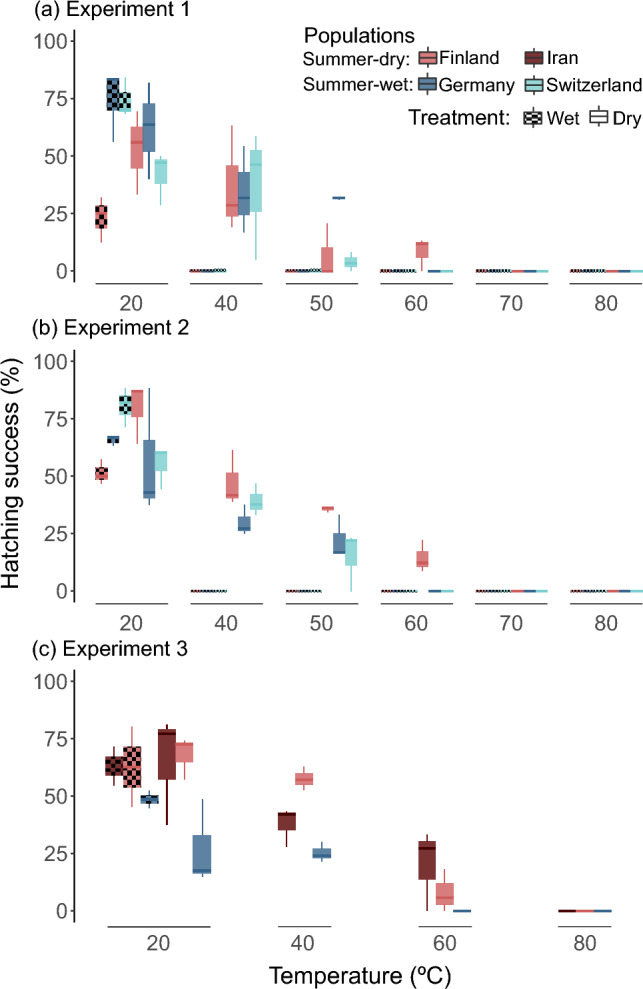
Hatching success per diapause treatment for **a** experiment 1 (N = 1980), **b** experiment 2 (N = 1953) and **c** experiment 3 (N = 927). Colours represent distinct *D. magna* populations and habitats of origin. Note that the Swiss population was only used in the experiments 1 and 2, and the Iranian population was only used in experiment 4. Wet and dry treatments are differentiated by a dotted or null pattern, respectively. Boxplots show the median, first, and third quantile of a technical replicate. Whiskers extend to 1.5 times the interquartile range upper and lower limits

**Fig. 3:**
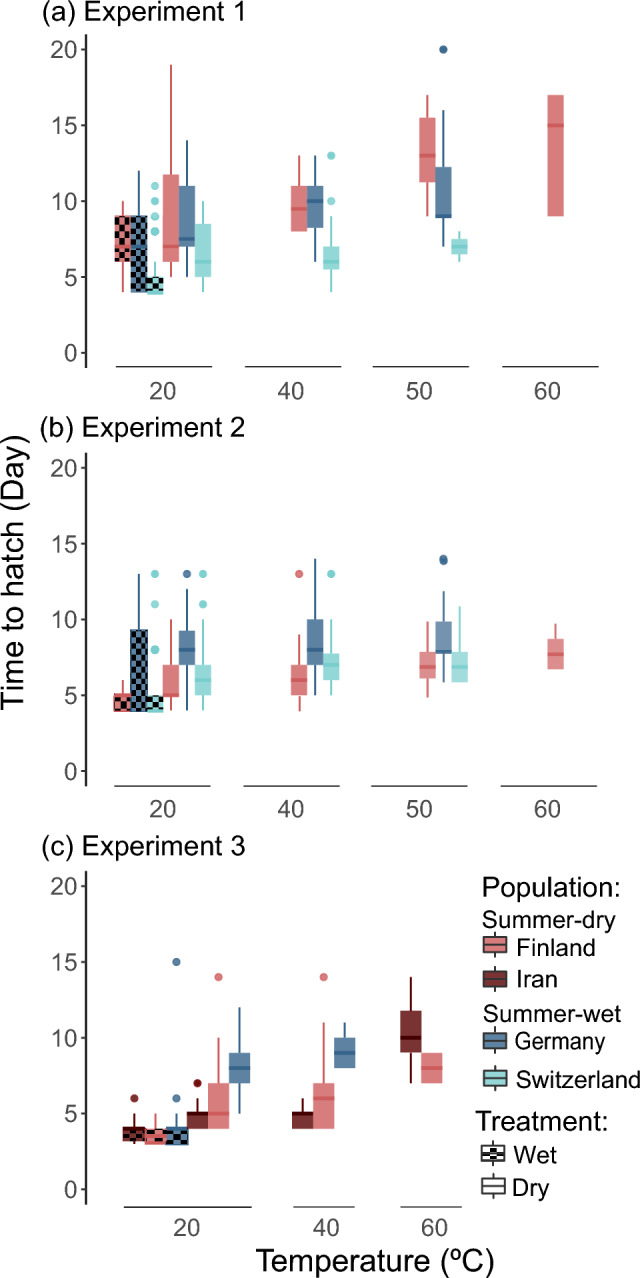
Time to hatch (days until hatching after induction) per diapause treatment for **a** experiment 1 (N = 269), **b** experiment 2 (N = 331) and **c** experiment 3 (N = 256). Colours, patterns and boxplots are explained in Fig. 2

Hatching success and time to hatch were variable among treatments, populations (experiments 1 to 3) and genotypes (experiment 4), but with a general pattern. No hatchlings were observed at 70 and 80 °C, regardless of the treatment (wet or dry), nor at temperatures above 20 °C in wet treatments (experiments 1 and 2) (Table 1, Fig. 2a and b). Reducing the duration of diapause treatments from 20 days in experiment 1 to 11 days in experiment 2 did not change results significantly, most notably for wet treatments at elevated temperatures (Table 2, Figs. 2a and b, 3a and b). In treatments where at least one hatchling was observed, hatchlings started appearing on day 3 or 4 and continued at a declining rate for 10 to 16 days after hatching induction (experiment 1).

**Fig. 4:**
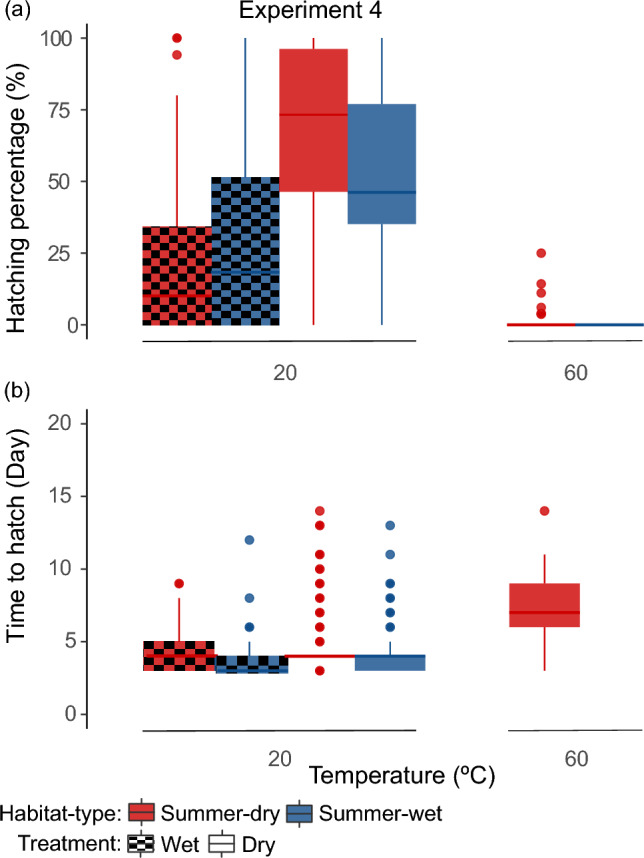
**a** Hatching success (N = 3398) and **b** time to hatch—days until hatching after induction—(N = 1154) per diapause treatment in experiment 4. Colours, patterns and boxplots are explained in Fig. 2

Higher temperatures during diapause consistently decreased hatching success and increased time to hatch across all four experiments and samples (Table 2, Figs. 2, 3, 4). This effect was independent of geographical origin or diapause condition (tested in experiment 1 and 2 for hatching success) (Table 2, Figs. 2, 3, 4). Although, temperature negatively affected hatching success after both wet and dry diapause, the combined effect of temperature and condition was more extreme in the wet treatments, where no hatchlings were observed at 40 °C or above for any population (i.e., experiment 1 and 2) (Table 1, Fig. 2a and b).

Wet diapause conditions resulted in lower hatching success (only significant for experiments 2 and 4) but in earlier hatching (at 20 °C) compared with dry treatments (Table 2, Figs. 2, 3, 4). We also found that hatching success in experiments 1, 2 and 4 was higher after a dry diapause (at any temperature) for resting stages from summer-dry habitats, while resting stages from summer-wet habitats performed better after a wet diapause at 20 °C (Table 2, Figs. 2 and 4a). In experiment 3, resting stages from the summer-wet population generally had lower hatching success than resting stages from the two summer-dry populations exposed to different conditions (Table 2, Fig. 2c).

All four experiments, consistently showed that resting stages from summer-wet habitats hatched only up to 50 °C after a dry diapause, while some resting stages from summer-dry populations (Experiments 1 to 3) or genotypes (Experiment 4) hatched successfully after a 60 °C diapause treatment (Table 1, Figs. 2 and 4). The individual effect of habitat type in hatching success was considered significant in experiments 1 to 3 (Table 2). However, due to the experimental design–with three populations per experiment–this result should be interpreted cautiously (Table 1).

**Fig. 5:**
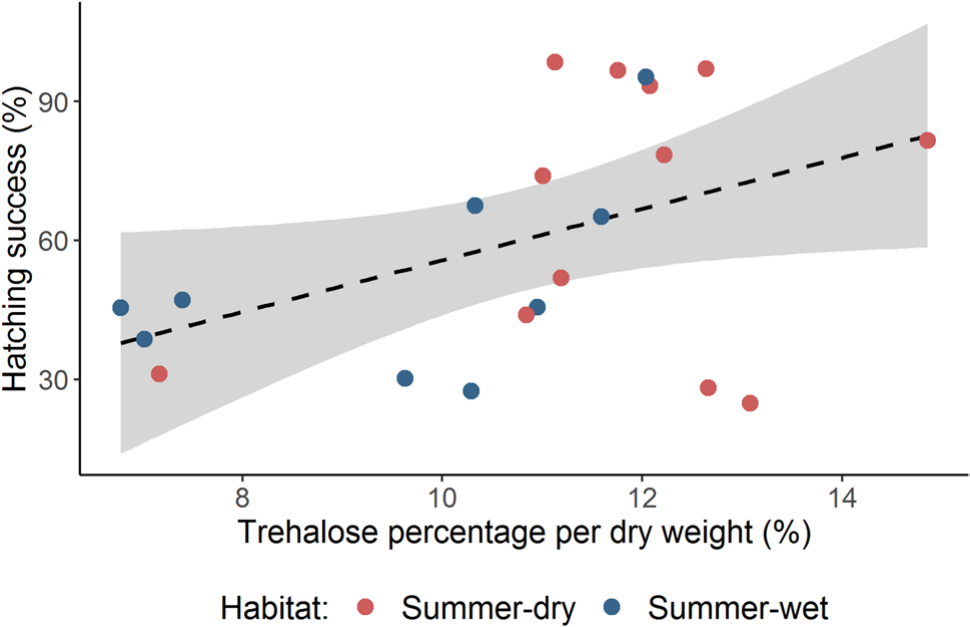
Variation in hatching success related to percentage of trehalose per resting egg dry weight for 21 *D. magna* genotypes used in experiment 4 (*see* Table SS1). Hatching success was estimated from the 20 °C dry diapause treatment (N = 1305) and averaged per genotype. Trehalose percentage per dry weight of resting eggs was retrieved from Santos and Ebert (2022). Each dot represents one genotype coloured according to habitat type. The dashed black line represents the linear regression model, including the 95% standard error of the mean in grey (LRM: Adjusted-R^2^ = 0.16, DF = 19, F-statistics = 4.84, p value = 0.04, intercept = 8.59, slope = 0.04)

In experiment 4, hatching success was highly variable among genotypes. Specifically, the genotype factor accounted for 72.6% and 74.2% of the variance in the 20 °C wet and dry treatments, respectively. Genotypes with higher trehalose content in their resting stages had a higher hatching success after a 20 °C dry diapause (Df = 19, F-statistic = 4.8, R^2^(adjusted) = 0.16, *p* value = 0.04) (Fig. 5), but not after a 20 °C wet diapause (Df = 19, F-statistic = 1.7, R^2^(adjusted) = 0.03, *p* value = 0.20).

## Discussion

Diapause allows organisms to survive harsh environmental conditions. Here, we tested how different populations and genotypes of *D. magna*, a pond-dwelling freshwater crustacean, fare under various diapause conditions. The two traits studied—hatching success and time to hatch—influence the dynamics of natural populations and determine their survival and are, in turn, strongly influenced by environmental conditions during diapause. These effects vary among populations and genotypes depending on their habitats.

When resting stages were kept wet during diapause, the negative effects of temperature were strong, with hatchlings recorded only at 20 °C, never at 40 °C or higher. This finding suggests that wet diapause at elevated temperatures represents a more stressful condition than dry diapause. Because waterbodies in the Holarctic climate zone generally do not reach temperatures above 40 °C (Seefeldt and Ebert 2019), the ability to deal with hot, wet diapause conditions may never have been selected for in the natural populations used in this study.

In contrast, warm, dry conditions are commonly encountered. Many freshwater habitats dry up during prolonged periods without rain, for example in the Mediterranean basin or where waterbodies are very small, such as rock pools. Under these conditions, resting stages of pond dwelling organisms experience elevated temperatures in the dry sediments (Yampolsky et al. 2014; Seefeldt and Ebert 2019). Our findings correspondingly show that the combined effect of dryness and heat is less harmful than wet heat, although, here too, we observed a decrease in hatching success and an increase in time to hatch for all populations and genotypes with elevated temperatures. In diapause treatments with extreme temperatures (above 60 °C), no hatchlings were observed. Elevated temperatures and drought are known to delay and inhibit seed germination in plants (Liu et al. 2019; Suriyasak et al. 2020), and development in some animal species after dormancy (Higaki and Ando 2002; Nielsen et al. 2022). Although extreme temperatures occur in nature (e.g., Zhao et al. 2021), mainly in dark and sun-exposed habitats, they have never been recorded for a *D. magna* population. Likewise, the absence of hatchlings after being exposed to those extreme temperatures again suggests that this species may only be adapted to a range of local conditions and that the future stability of some populations might depend on how fast they can adapt to the rate of temperature increases.

We hypothesized that elevated temperatures and drought would impact the resting stages of *D. magna* genotypes from summer-dry habitats less than those from summer-wet habitats. This was consistently shown in our first three experiments where only some resting stages collected from the natural sediments of summer-dry populations hatched in the dry, 60 °C diapause treatments. A fourth experiment using resting stages produced by individual genotypes to exclude the possible effects of maternal, age or environmental factors also corroborated this hypothesis. In it, only resting stages from summer-dry genotypes hatched after a dry diapause treatment of 60 °C. We also found that resting stages from summer-dry habitats, where sediment temperatures can exceed 50 °C when ponds dry up (Seefeldt and Ebert 2019), performed better in dry conditions. These findings indicate that populations from summer-dry habitats impose strong natural selection on the stress tolerance of resting stages and are thus locally adapted to these conditions. It should be noted that although the effect of habitat type was rather weak, a pattern of local adaptation was strong enough to be observed in all four experiments.

A possible mechanism used by *Daphnia* resting stages to survive stressful conditions is to produce trehalose, a natural sugar known for conferring resistance to drought and heat in different life stages, including dormant plant seeds and animal eggs (Crowe et al. 1998; Kosar et al. 2019; Huang et al. 2021). Santos and Ebert (2022) found that *D. magna* resting stages from summer-dry habitats contain more trehalose than those from summer-wet habitats. Here, we extend this finding by showing that hatching success of dry diapausing resting stages is positively correlated with trehalose content in the eggs. Thus, the presence of more trehalose may allow resting stages to survive better under conditions of drought. This investment in drought resistance possibly entails costs, however, or else resting stages from all populations would may contain high amounts of trehalose.

In experiment 4, hatching success at 20 °C was lower than the first three experiments and than previous laboratory experiments (e.g., (De Meester 1993)). A major difference between the resting stages from natural sediments used in the first three experiments and the ones used in the fourth experiment was that the natural sediment samples were likely mostly outbred, whereas the resting stages produced by the genotypes in experiment 4 resulted from selfing, i.e., clonally produced sons fertilize their clonal sisters. Such inbreeding is known to reduce hatching success (Innes 1989; De Meester 1993). In addition, the laboratory-produced resting stages had a shorter diapause, which can also reduce hatching success (Ślusarczyk et al. 2019). Also, the age, origin, season of production, diapause length and conditions of its termination were all unknown for the field-collected resting stages, and diverse genotypes were present in the population samples. All these factors can contribute to within-population variation for each treatment (Moritz 1987; De Meester 1993; Ślusarczyk et al. 2019). In fact, in experiment 4, the genotype effect was responsible for a large variation in hatching success.

Hatching delay was longer after dry diapause than wet diapause treatments, suggesting that resting eggs that have been dry for an extended time need longer to re-activate their development. Trehalose seems not to be present in planktonic individuals at ambient conditions (*personal observations*), and it is considered the only respiratory substrate for diapause emergence in *Artemia salina* (Clegg 1964). This may also be the case in *D. magna*. Elevated osmotic pressure and water deficiency correlate with reduced oxygen consumption and trehalose oxidation rates, so that reaching the energetic requirements for development after diapause takes longer, thus increasing time to hatch (Clegg 1964).

## Conclusion

For many organisms, life in habitats with strong temporal and environmental variation is only possible by forming resting stages to endure stressful conditions. However, these resting stages are also challenged under stressful environmental conditions. Our experiments seek to understand the limits of successful diapause, uncovering the surprising result that the combination of two stress factors (dryness and heat) is less harmful than heat alone for the planktonic crustacean *D. magna*. Of the treatment combinations examined in this study (dry vs. wet, and ambient vs. high temperatures), wet-ambient and dry-warm conditions are those predominantly found in nature (Roulin et al. 2013; Seefeldt and Ebert 2019). Consequently, natural selection has not produced phenotypes to deal with other combinations, in particular, wet and warm conditions. The finding that organisms from habitats with a high propensity to dry up in summer seem to perform consistently better in warm, dry diapause conditions supports this hypothesis. A crucial element in the stress resistance of many organisms is trehalose, which is known to be present in resting eggs and which is found in greater amounts in resting stages from summer-dry *D. magna* populations (Santos and Ebert 2022). The *Daphnia* system may provide us an opportunity to explore the underlying genetics of trehalose’s role, using genomic association studies to explore its costs and benefits. Although such studies have been done in bacteria and plants (McIntyre et al. 2007; Dan et al. 2021), they have not yet been done in animals. Lastly, considering the foreseeable increase in temperature and droughts in temperate regions over the upcoming years, it is vital to understand the mechanisms that keystone species such as *D. magna* use to cope with extreme diapause conditions in order to predict their response and help maintain the equilibrium of freshwater ecosystems.

### Supplementary Information

Below is the link to the electronic supplementary material.

Supplementary file1 (PDF 264 KB)

## Data Availability

The datasets produced in this study and used for analysis are available at doi: https://doi.org/10.6084/m9.figshare.21806568.

## Code availability

The scripts used in this study are available in doi: https://doi.org/10.6084/m9.figshare.21806583.
