# Supplementary material for: The limits of stress-tolerance for zooplankton resting stages in freshwater ponds
Source: Oecologia. 2023 Nov 16;203(3-4):453–65. doi: 10.1007/s00442-023-05478-8 (PMC10684647; doi:10.1007/s00442-023-05478-8)
Supplement: Supplementary file 1 — Supplementary file1 (PDF 264 KB) [file 442_2023_5478_MOESM1_ESM.pdf]

Title:

The limits of stress-tolerance for zooplankton resting stages in freshwater ponds

Authors

Joana L. Santos[1] & Dieter Ebert[1]

[1]Department of Environmental Sciences, Zoology, University of Basel, Vesalgasse 1, 4051 Basel, Switzerland

orcID:

0000-0003-2939-7091 (J.L.S.)

0000-0003-2653-3772 (D.E.)

Corresponding author

Joana L. Santos

Email: joana_santos222@hotmail.com

Telephone: 0041 61 2070372

Author Contributions: JS and DE designed the study; JS performed the laboratory experiments; JS and DE performed the analysis; JS wrote the manuscript and DE revised it; DE provide the funding for the study.

Supplementary Material

Table SS1. Description of genotypes used in experiment 4 and their habitats of origin. Asterisks indicate genotypes removed from analysis due to no or very few observed hatchlings. [t] indicates genotype used for correlation of trehalose content (*retrieved from* Santos and Ebert, 2022) and hatching success.

**Genotype**	**Country**	**Habitat type**
BE-HO-1*	Belgium	summer-wet
BY-G-9 [t]	Belarus	summer-wet
CH-H-1 [t]	Switzerland	summer-wet
CY-PA2-1*	Cyprus	summer-dry
CY-PA3-1*	Cyprus	summer-dry
CZ-KO-1 [t]	Czech Republic	summer-wet
DK-RL-3 [t]	Denmark	summer-wet
ES-D-BDE1*	Spain	summer-dry
ES-HT-1 [t]	Spain	summer-dry
FI-FAT-1-3*	Finland	summer-dry
FI-FSP1-16-2*	Finland	summer-dry
FI-FUT1-2-1 [t]	Finland	summer-dry
FI-OER-3-3 [t]	Finland	summer-dry
FI-SK-58-2 [t]	Finland	summer-dry
FI-SKW-2-1 [t]	Finland	summer-dry
FR-C1-1	France	summer-dry
GB-C1-1*	Gran-Britain	summer-wet
GB-EK1-1*	Gran-Britain	summer-wet
GB-EK2-6 [t]	Gran-Britain	summer-wet
GB-FML-1 [t]	Gran-Britain	summer-wet

GB-S17-7	Gran-Britain	summer-wet
HU-AG-03*	Hungary	summer-dry
IE-DUB-1*	Ireland	summer-wet
IL-BM-1*	Israel	summer-dry
IL-M1-8*	Israel	summer-dry
IT-MDV-1*	Italy	summer-dry
IT-PER-2 [t]	Italy	summer-dry
MA-ES-3*	Morocco	summer-dry
NO-AA-1 [t]	Norway	summer-wet
NO-F-1*	Norway	summer-wet
NO-LADE-1*	Norway	summer-wet
NO-RO-1 [t]	Norway	summer-wet
RU-BN-BB6*	Russia	summer-wet
RU-BOL1-1 [t]	Russia	summer-dry
RU-C10-06*	Russia	summer-dry
RU-KOR1-1 [t]	Russia	summer-dry
RU-MA4-3*	Russia	summer-dry
RU-R2-1 [t]	Russia	summer-wet
RU-VOL-36*	Russia	summer-dry
SE-BY-J6	Sweden	summer-wet
SE-G1-9 [t]	Sweden	summer-dry
SE-GN2-3A10 [t]	Sweden	summer-dry
SE-H1-1 [t]	Sweden	summer-dry
UA-KR-1-7 [t]	Ukraine	summer-dry

Figure SS1. Detailed information of model selection for time to hatch dependent variable. Densities histograms of observed data and fitted curves for the Poisson (in red) and negative binomial (in green) model distribution, based on the *fitdistr* function from the MASS v5.3-54 (Venables and Ripley 2002) R package, are shown in a) to d) for experiment 1 to 4, respectively. The Cullen and Frey graphs obtained from the *descdist* function from the fitdistrplus v1.1-11 R package (Delignette-Muller and Dutang 2015), including a bootstrap of 1000 are presented in the upper-left of a) to d). In e) an overview of data mean and variance, the comparisons of the AIC parameters for data distribution and models using Poisson and negative binomial (n_binomial) distribution are shown for each experiment, and the *p*-value associated to the LTR comparison between the two models. Negative binomial distribution was implemented using *glm.nb* function from the MASS v5.3-54 (Venables and Ripley 2002) R package, from which the theta parameter was retrieved. LTR comparison was implemented using *simulateLRT* function from the DHARMa V0.4.6 (http://florianhartig.github.io/DHARMa/) R package. The hypotheses that the model with a negative binomial distribution is better than with a poisson distribution was not supported for any experiments. Dispersion was tested using *testDispersion* function of the same package, and it was significant for experiments 2 to 4 in models including both Poisson and negative binomial distributions. The combination of the evaluated parameters and the observed small difference between mean and variance prompted us to apply a Poisson error distribution in the Generalized Linear Mixed Models (GLMM). Due to the considerable underdispersion detected in the data from experiment 4, the statistics of the GLMM model implementing a negative binomial error distribution are presented in f) for comparison. We highlight that for experiment 4, both models using Poisson and negative binomial distribution recovered similar results.

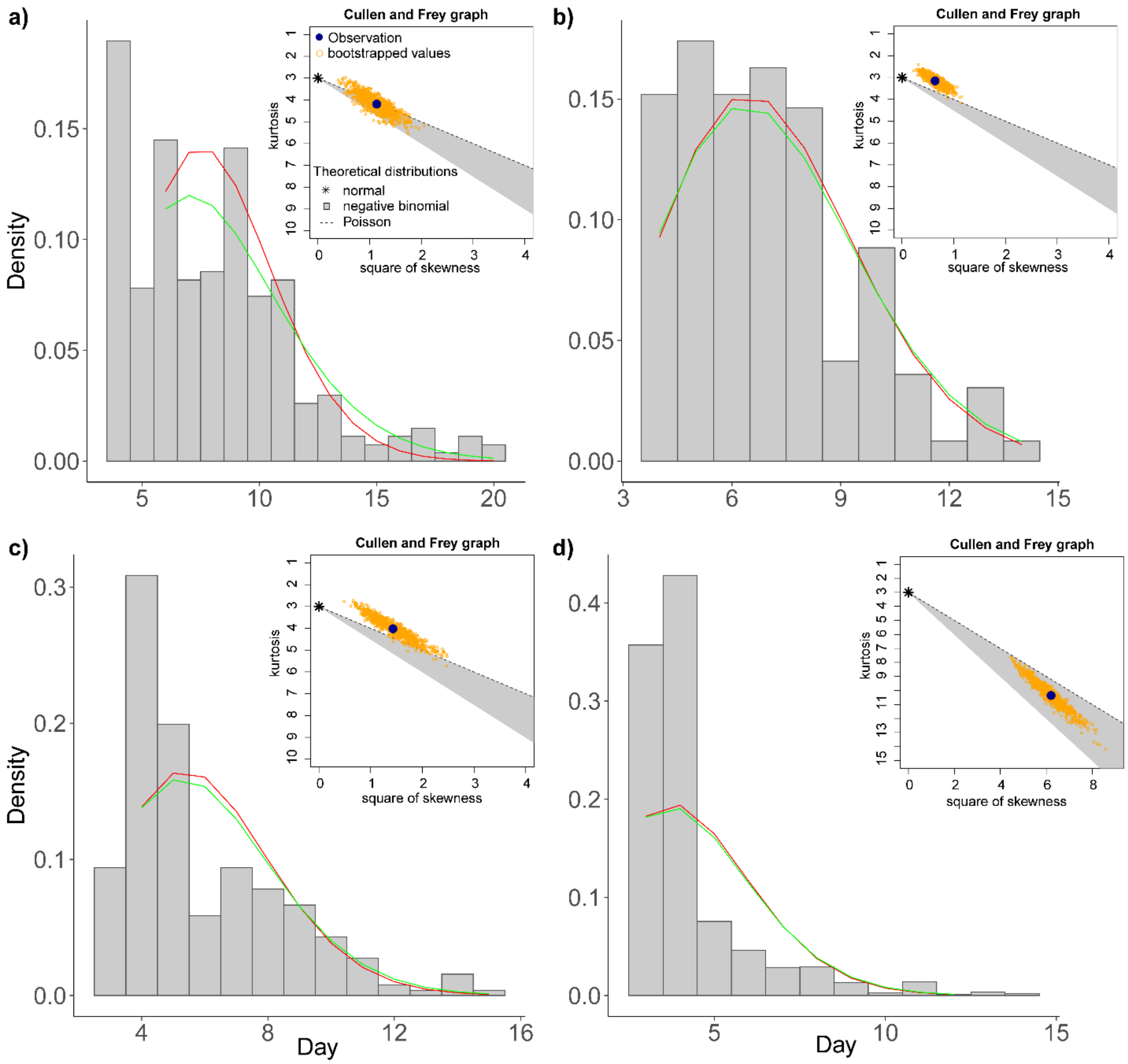

e)

Experiment	Mean	Variance	Data Distribution		AIC GLMM Model			LRT comparison *H1: n_binomial better than Poisson model*
			Poisson AIC	n_binomial AIC	Poisson AIC	n_binomial		
						Theta	AIC	
**Exp. 1**	8.01	12.25	1421	1399	1295	295585.5	1297	*p*=0.916
**Exp. 2**	6.96	5.62	1638	1640	1589	497225.4	1591	*p*=1
**Exp. 3**	5.89	6.46	1168	1169	1035	509337.6	1037	*p*=1
**Exp. 4**	4.25	2.89	4414	4416	4293	441795.4	4295	*p*=1

f)

Time to Hatch – Experiment 4 – Negative binomial distribution model

Variables	Df	χ^2	*p*-value
Temperature	1	22.96	1.69×10^{-6}
Condition	1	3.96	0.047
Habitat	1	1.95	0.162
Condition * Habitat	1	0.02	0.885
Population	1	87.18	$<2.2\times10^{-16}$
Technical replicate	1	0	1
